# Impact of Lesion Delineation and Intensity Quantisation on the Stability of Texture Features from Lung Nodules on CT: A Reproducible Study

**DOI:** 10.3390/diagnostics11071224

**Published:** 2021-07-06

**Authors:** Francesco Bianconi, Mario Luca Fravolini, Isabella Palumbo, Giulia Pascoletti, Susanna Nuvoli, Maria Rondini, Angela Spanu, Barbara Palumbo

**Affiliations:** 1 Department of Engineering, Università Degli Studi di Perugia, Via Goffredo Duranti 93, 06135 Perugia, Italy; mario.fravolini@unipg.it (M.L.F.); giulia.pascoletti@polito.it (G.P.); 2 Section of Radiation Oncology, Department of Medicine and Surgery, Università Degli Studi di Perugia, Piazza Lucio Severi 1, 06132 Perugia, Italy; isabella.palumbo@unipg.it; 3 Department of Mechanical and Aerospace Engineering (DIMEAS), Politecnico di Torino, Corso Duca Degli Abruzzi, 24, 10129 Torino, Italy; 4 Unit of Nuclear Medicine, Department of Medical, Surgical and Experimental Sciences, Università Degli Studi di Sassari, Viale San Pietro 8, 07100 Sassari, Italy; snuvoli@uniss.it (S.N.); maria.rondini01@ateneopv.it (M.R.); aspanu@uniss.it (A.S.); 5 Section of Nuclear Medicine and Health Physics, Department of Medicine and Surgery, Università Degli Studi di Perugia, Piazza Lucio Severi 1, 06132 Perugia, Italy; barbara.palumbo@unipg.it

**Keywords:** computed tomography, texture features, lung nodules, radiomics, lesion delineation, intensity quantisation, stability

## Abstract

Computer-assisted analysis of three-dimensional imaging data (*radiomics*) has received a lot of research attention as a possible means to improve the management of patients with lung cancer. Building robust predictive models for clinical decision making requires the imaging features to be stable enough to changes in the acquisition and extraction settings. Experimenting on 517 lung lesions from a cohort of 207 patients, we assessed the stability of 88 texture features from the following classes: first-order (13 features), Grey-level Co-Occurrence Matrix (24), Grey-level Difference Matrix (14), Grey-level Run-length Matrix (16), Grey-level Size Zone Matrix (16) and Neighbouring Grey-tone Difference Matrix (five). The analysis was based on a public dataset of lung nodules and open-access routines for feature extraction, which makes the study fully reproducible. Our results identified 30 features that had good or excellent stability relative to lesion delineation, 28 to intensity quantisation and 18 to both. We conclude that selecting the right set of imaging features is critical for building clinical predictive models, particularly when changes in lesion delineation and/or intensity quantisation are involved.

## 1. Introduction

Lung cancer, excluding skin cancer, is the second most common type of cancer in both genders after prostate cancer in men and breast cancer in women [1,2]. Unfortunately, the overall five-year survival rate of patients with lung cancer is still dismally low (≈18.6%) and far below that of the other types of oncological disorders such as colorectal (≈64.5%), breast (≈89.6%) and prostate (≈98.2%) cancer [2]. The survival rate, however, depends a great deal on the stage of the disease when it is first diagnosed, ranging from a grim ≈5% for distant tumours to ≈56% when the disease is still localized [2].

The effectiveness of lung cancer therapy strongly relies on early diagnosis. Chest radiographies (CXRs), computed tomography (CT), magnetic resonance imaging (MRI), positron emission tomography (PET), cytology sputum and breath analysis represent the currently available detection techniques for lung cancer [3]. Computed tomography, in particular, plays a pivotal role in differentiating benign vs. malignant lung nodules in the early screening phase. However, although CT scans provide valuable information about suspicious lung nodules, their correct interpretation can be a challenging task for the radiologist. In this context, computer-assisted diagnosis may provide a valid support for the radiologist to contribute to the diagnostic process of lung cancer.

**Figure 1:**
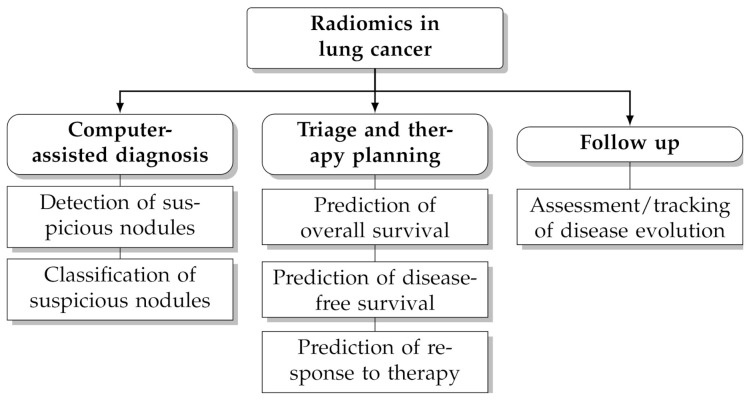
Potential applications of radiomics in lung cancer.

In recent years computerised analysis of imaging data (particularly from CT and PET/CT) has shown great promises to improve the management of patients with lung cancer [4,5,6,7,8,9,10]. The rationale behind this paradigm is that the quantitative extraction of imaging parameters from suspicious lesions—particularly shape and texture features—may reveal hidden patterns that would otherwise go unnoticed to the naked eye [11,12]. Furthermore, the extraction of objective, reproducible and standardised imaging parameters helps reduce the intra-observer and inter-observer bias and facilitates tracking changes over time. Radiomics leverages on artificial intelligence techniques and the increasing availability of large, open-access and multicentric datasets of pre-classified cases to infer clinical information about unknown ones (‘population imaging’ approach [13]). Several studies have underlined the potential benefit of radiomics for clinical problem-solving in lung cancer, such as prediction of malignancy [14,15,16], histological subtype [17,18,19], prognosis [20,21,22] and response to treatment [23,24,25] (see also Figure 1 for an overview of potential applications).

The radiomics pipeline consists of six steps [26]: (1) acquisition, (2) pre-processing, (3) segmentation (also referred to as delineation), (4) feature extraction, (5) post-processing and (6) data analysis/model building. The fourth step, which aims at extracting a set of quantitative parameters from the region of interest, is central to the whole process and various studies have shown that steps 1–3 can have significant impact on feature extraction [27,28,29,30,31,32]. A current major research focus is therefore the assessment of the stability of radiomics features to changes in image acquisition settings, signal pre-processing and lesion delineation (see Traverso et al. [33] for a general review on the subject).

In particular, the repeatability and reproducibility of radiomics features from lung lesions on CT has been investigated in a number of recent works. In [34] Balagurathan et al. evaluated the test-retest reproducibility of texture and non-texture features from chest CT scans to two consecutive acquisitions (on the same patient) taken within 15 min from one another. Of the 329 features included in their study, they found that 29 (i.e., approximately one in eleven) had a concordance correlation coefficient (CCC) >0.9. The study by Seung-Hak et al. [35] addressed the impact of voxel geometry and intensity quantisation on 260 lung nodules at CT; in this case the results indicated that nine of the 252 features investigated had high reproducibility among the different experimental settings. As for stability to lesion segmentation, Kalpathy-Cramer et al. [36] investigated 830 radiomics features from CT scans of pulmonary nodules at CT and determined that 68% of them had CCC≥0.75. Parmar et al. [37] compared the variability of radiomics features extracted from automatically segmented lesions (3D-Slicer) with that of features from manually-segmented ones and found higher reproducibility in the first case. Owens et al. [38] examined the repeatability of 40 radiomics features from ten CT scans of non-small lung cancer to manual and semi-automatic lesion delineation consequent to intra-observer, inter-observer and inter-software variability. Similarly to [37], they concluded that semi-automatic lesion delineation can provide better reproducible radiomics features than manual segmentation. Tunali et al. [39] assessed the repeatability relative to the test-retest of 264 radiomics features from the peritumoural area of lung lesions and their stability to nine semi-automated lesion segmentation algorithms. They determined an unlikely response between the different classes of texture features investigated with first-order features generally showing better stability than the other groups. More recently, Haarburger et al. [40] evaluated the stability of 89 shape and texture features to manual and automatic lesion delineation, finding that 84% of the features investigated had intra-class correlation coefficient (ICC) >0.8.

One common shortcoming in the available studies, however, is that most of them are based on proprietary datasets (with the notable exception of [40]) and custom feature extraction routines, all of which renders the results difficult to reproduce. In this work we investigated the stability of 88 textural features from CT scans of lung lesions to delineation and intensity quantisation. To guarantee reproducibility we based our study on a public dataset (LIDC-IDRI [41]) and on feature extraction routines from an open-access package (PyRadiomics [42]). Furthermore, we made all the code used for the experiments freely available to the public for future comparisons and evaluations. Our results identified 30 features that had good or excellent stability to lesion delineation, 28 to intensity quantisation and 18 to both.

## 2. Materials and Methods

### 2.1. Patient Population

The study population included a total of 517 lung lesions (axial diameter = 16.2 ±11.5 mm (3.2–72.3 mm)) from a cohort of 207 patients (110 males, 97 females, age = 59.0 ± 14.7 year (14–88 year)) who underwent thoracic computed tomography (CT) for lung cancer screening. The data were sourced from the open-access Lung Image Database Consortium collection (LIDC-IDRI [41,43]). Since this is a multicentric dataset, different imaging systems, acquisition protocols and image reconstruction settings were used and included among them are the following: tube voltage 120–140 kV, in-plane pixel spacing 0.53–0.98 mm, slice thickness 0.6–3.0 mm and slice spacing 0.5–3.0 mm. Each nodule was manually annotated by one to five radiologists relative to subtlety (difficulty of detection), internal structure (soft, fluid, fat or air), pattern of calcification (if present), sphericity, margin (sharp vs. poorly defined), degree of lobulation, extent of spiculation, radiographic solidity (solid, non-solid, ground-glass or mixed) and subjective assessment of the likelihood of malignancy (from highly unlikely to highly suspicious). The complete (anonymous) list of the patient IDs along with the main acquisition settings for each scan and that of each nodule with the related annotations are provided as Supplementary Material (scans_metadata.csv, nodules_metadata.csv). Further details about the study population can also be retrieved from the LIDC-IDRI repository either through the pylidc interface or via direct access to the DICOM data (see also Section 2.5 on this point). Scans with incomplete metadata (e.g., lack of patient’s gender and/or age were excluded from our study).

### 2.2. Image Preprocessing

Pre-processing involved uniform intensity discretisation within a fixed-width window. The original CT signal was first clipped between ${\mathrm{CT}}_{min}=\left(\mu_{d}-2\sigma_{d}\right)$ and ${\mathrm{CT}}_{max}=\left(\mu_{d}+2\sigma_{d}\right)$, where $\mu_{d}$, $\sigma_{d}$, respectively, represent the mean and standard deviation of the average nodule density in the dataset. The resulting bounds were ${\mathrm{CT}}_{min}$ = −583 HU and ${\mathrm{CT}}_{max}$ = 137 HU (window level = −223 HU, width = 720 HU). Intensity values below the lower bound or above the upper bound were set to ${\mathrm{CT}}_{min}$ or ${\mathrm{CT}}_{max}$, respectively. Uniform signal quantisation was then applied using $N_{g}=$ 32, 64, 128 and 256 discretisation levels, which in terms of bin width corresponded to approximately 23 HU, 11 HU, 6 HU and 3 HU, respectively. No further pre-processing operations such as filtering or spatial resampling/interpolation were applied.

### 2.3. Feature Extraction

A total of 88 textural features from six classes were included in this study (see Table 1 and Table 2 for the complete list). All the features are compliant with the Imaging Biomarker Standardization Initiative (IBSI [44]); volume-confounded features were not considered in the analysis. For mathematical definitions and formulae we refer the reader to the documentation available in [42]. Grey-level co-occurrence matrix (GLCM), grey level dependence matrix (GLDM) and Grey level size zone matrix (GLSZM) features were all computed using inter-voxel distance δ=1 and a three-dimensional 26-connectivity voxel neighbourhood. Feature extraction was based on the open-source PyRadiomics package (see also Section 2.5 for further details).

### 2.4. Experimental Design and Stability Assessment

**Figure 2:**
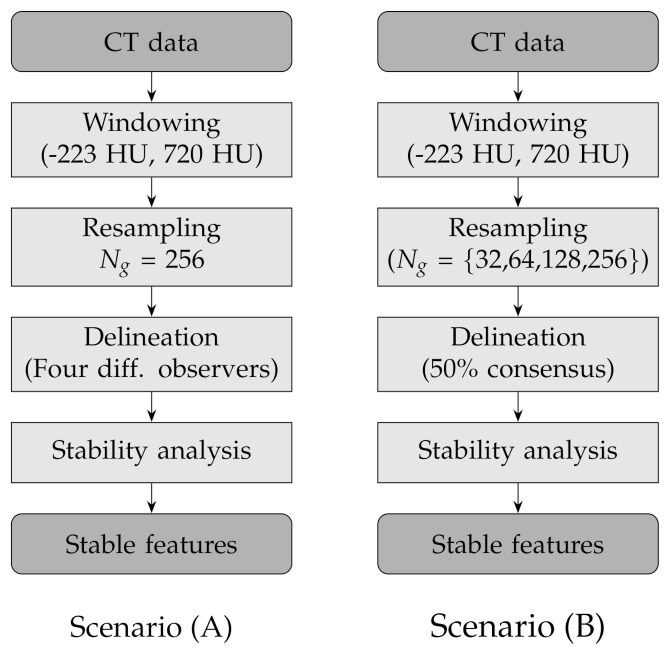
Flow charts of the experimental design for the two scenarios: lesion delineation (scenario **A**) and intensity resampling (scenario **B**).

In order to assess the stability of the texture features to lesion delineation (A) and intensity resampling (B), we considered two experimental scenarios with the following combinations of factors (see also Table 3 and Figure 2 for a round-up):

(A) Fixed number of quantisation levels ($N_{g}=256$) and four lesion delineations per nodule; each delineation was generated by one different observer;
(B) Number of quantisation levels for intensity resampling $N_{g}=$ 32, 64, 128 and 256 and fixed lesion delineations based on consensus consolidation at 50% agreement level—that is, a voxel was considered in the lesion when at least 50% of the available delineations included that voxel and not in the lesion otherwise.

**Figure 3:**
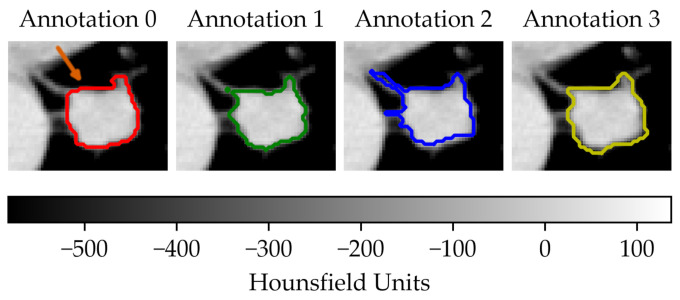
Sample of a lung lesion and four manually delineated boundaries. Each annotation was generated by a different observer. The orange arrow indicates the region of interest.

**Figure 4:**
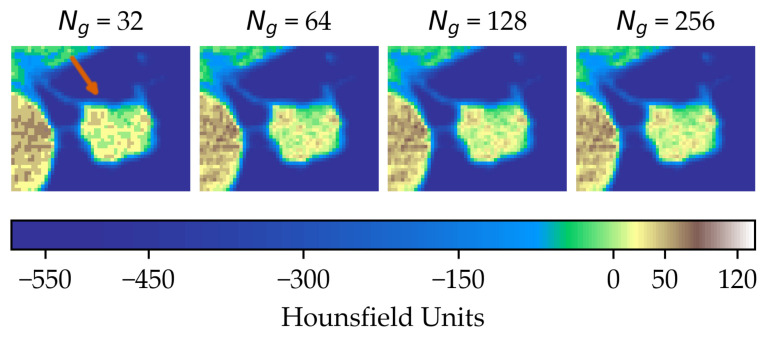
Effect of intensity resampling. Observe the difference in the texture granularity (subtle but noticeable) particularly between $N_{g}=32$ and $N_{g}=64$. The orange arrow indicates the region of interest.

In the first scenario (A) we limited the analysis to a subset of 206 nodules from 130 patients for which four lesion annotations were available for each nodule (see Figure 3 for an example of different delineation on the same nodule). The observers (raters) are unknown and, due to the multicentric nature of the dataset, they are likely to be different from one nodule to another. In the second scenario (B), we had four different (but fixed) quantisation levels, which can be considered equivalent to different raters (Figure 4).

In both cases the assessment of feature stability was based on the average Symmetric Mean Absolute Percentage Error (sMAPE [45,46]). Specifically, for each nodule and set of raters (delineation or intensity resampling), we computed the average sMAPE for all the observation pairs and averaged the results over the whole population. In formulas, denoted with $x_{ij}$ the reading on the *i*-th nodule by the *j*-th rater the by-nodule sMAPE $S_{i}$ is defined as follows:

(1) $$S_{i}=\frac{1}{\left|P([J],2)\right|}\sum_{\left(f,t)\in P([J],2\right)}\frac{\left|{\hat{x}}_{i}-x_{i}\right|}{\left|{\hat{x}}_{i}\right|+\left|x_{i}\right|}\times 100$$

where *J* is the number of raters and P([J],2) denotes the 2-ordered subsets of [J], that is, all the pairwise permutations of {1,⋯,J}. In other words, $S_{i}$ computes the average sMAPE between pairs of readings each given by two observers, where one of the observers is being alternatively considered the reference (therefore returning the ‘true’ value $x_{i}$) or the estimator (giving the “forecast” value ${\hat{x}}_{i}$). One advantage of $S_{i}$ is that it counteracts the intrinsic asymmetry—despite its name—of the sMAPE [46]. Furthermore, since it is customary in the practice [45], we omitted the division by two from the summand’s denominator in Equation (1), which forces $S_{i}$ to have values in [0,100]. Finally, we obtained the overall stability measure *S* by averaging $S_{i}$ over all the nodules:

(2) $$S=\frac{1}{N}\sum_{i=1}^{N}S_{i}$$

where *N* is the total number of nodules. For an easier interpretation of the results we established a qualitative scale of feature stability in four grades as detailed in Table 4. A discussion about the use of sMAPE compared with other stability measures is also presented in Section 4.

### 2.5. Implementation, Execution and Reproducible Research

The experiments were carried out on a laptop PC with Intel${}^{®}$ Core${}^{™}$ i7-9750H CPU @ 2.60 GHz, 32 GB RAM, NVIDIA Quadro T1000 (4 GB) graphics card and Windows 10 Pro 64-bit operating system. The implementation was based on Python 3.8.6, with functions from dicom-parser 0.1.6 [47], NumPy 1.18.5 [48], Pandas 1.1.3 [49,50], pylidc 0.2.2 [51,52], pynrrd 0.4.2 [53] and Py-Radiomics 3.0.1 [42,54]. For reproducible research purposes, all the code and settings are available on the following GitHub repository: https://github.com/bianconif/stability_radiomics_features_lung_ct, accessed on 3 July 2021.

## 3. Results

Table 5, Table 6, Table 7, Table 8, Table 9 and Table 10 summarise the results of the experiments. As it can be observed, of the 88 features considered in the study, 18 showed good or excellent stability (defined as S≤ 10%) relative to both lesion delineation and intensity quantisation. Broken down by class, the number (percentages) of features with at least good stability relative to both delineation and intensity quantisation were: 4/13 (≈31%) for first-order features, 6/24 (33%) for GLCM features, 1/14 (≈7%) for GLDM features, 5/16 (31%) for GLRLM features and 2/16 (≈13%) for GLSZM features, whereas none of the five NGTDM features achieved at least good stability relative to both conditions.

**Figure 5:**
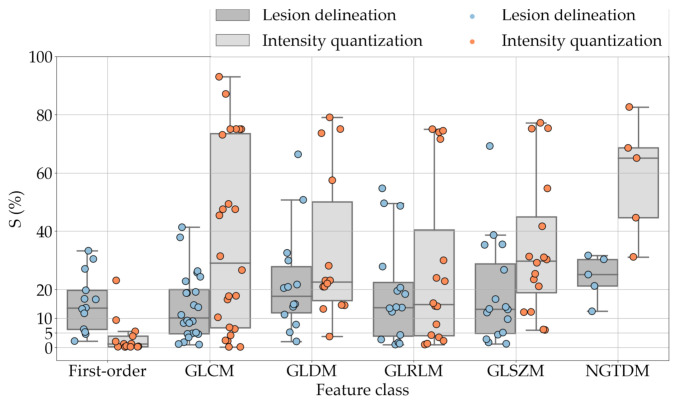
Stability of the texture features by class. Each dot represents one texture feature; the corresponding values are reported in Table 5, Table 6, Table 7, Table 8, Table 9 and Table 10.

If we examine the results by class of features we observe that those of the first-order (except Uniformity) all had at least good repeatability relative to intensity quantisation (this is evident also from Figure 5). This is, of course, what we expected, as these features (excluding Entropy and Uniformity) are by definition independent on signal quantisation—apart from numerical round-off errors. It is also no surprise that Entropy and Uniformity (Respectively defined as ‘Discretised intensity entropy’ and ‘Discretised intensity uniformity’ in the Image Biomarker Standardisation Initiative [55]) exhibited the highest relative error (9.40% and 23.05%, respectively), for they depend—by definition—on the number of quantisation levels used. Under the currently accepted formulations [42,55], Entropy and Uniformity have values in $\left[0,\log_{2}\left(N_{g}\right)\right]$ and $\left[1/N_{g},1\right]$, respectively, which sets into evidence the dependency on $N_{g}$. As for stability to lesion delineation, it emerged that Max was the most stable feature. This is coherent with tissue density being usually highest in the central area of the lesion, which is also the part of the tissue that most observers would include in the delineation. The other parameters that had good to excellent stability were Entropy, Range and Min.

For the other classes, Figure 5 indicates that the data about intensity quantisation were, on the whole, more dispersed than those about lesion resampling. Features from GLCM generally proved more resilient to changes in lesion delineation (half of them had S≤10.0) than intensity resampling (only seven features out of 24 reached at least good stability). This is, again, coherent with the GLCM definition depending heavily on the number of quantisation levels.

Similar arguments hold for the other classes of texture features. In particular, GLDM produced very few stable features: Only three of them showed at least good stability to lesion delineation and only one to intensity resampling. It is worth recalling that GLDM is based on the concept of ‘depending’ voxels [42,56]; that is, a neighbouring voxel is considered dependent on the central voxel if the absolute difference between the intensity values of the two is below a user-defined threshold α. For the threshold value we used the default PyRadiomics settings (α=0) and this may have had an effect—possibly negative—on the stability of this group of features. Likewise, GLSZM features were highly sensitive to signal quantisation too, which is again logical given the definition of GLSZM. Recall that this is based on sets of connected voxels (grey zones) sharing the same grey-level intensity; consequently, changes in signal quantisation are likely to produce different grey-zones, with fewer quantisation levels resulting in larger grey-zones and vice versa. This inevitably reflects on the feature values.

Notably, none of the NGTDM features proved resilient enough to both lesion delineation and intensity resampling (Table 10). As for lesion delineation, only Busyness and Strength attained excellent and good stability, respectively, whereas Coarseness was the only feature with good stability to intensity resampling. Consider that NGTDM [42,57] estimates the joint probability between the intensity level at one voxel and the average intensity difference among its neighbour voxels; we speculate that changing the number of resampling levels ($N_{g}$) may alter the joint distribution and this could explain the poor stability to signal quantisation.

## 4. Discussion

Radiomics has attracted increasing research interest in recent years as a possible means to assist physicians in clinical decision making. Potential applications in pulmonary imaging include, in particular, detection and assessment of suspicious lung nodules; prediction of histological subtype, prognosis and response to treatment. The radiomics workflow involves six steps, each of which is sensitive to a number of settings and parameters. Stability of radiomics features to these settings is therefore critical for guaranteeing reproducibility and consistency across multiple institutions.

Regarding stability to lesion delineation, a comparison with previously published works indicate that our results are by and large in agreement with what was reported by Haarburger et al. [40] concerning first-order, GLDM, GLSZM and GLRLM features. However, our study indicated lower stability of GLCM and NGTDM features than reported in [40]. One possible explanation of this discrepancy might be that the bin width used here (≈3 HU) was different than adopted in [40] (25 HU). In [34] Balagurunatan et al. found 29 features stable to lesion delineation, of which five were also investigated in the present work. Our findings show partial overlap with [34]: first-order Entropy and GLRLM RLN achieved good stability in both studies; on the other hand, GLCM Contrast, GLRLM GLN and RLN were stable in [34] but not here. As for intensity quantisation, Lee et al. [35] reported three highly stable (ICC >0.7) first-order features (Max, Min and Entropy), which confirmed their performance (S≤10%) in our experiments, and two GLCM features (DiffEnt and Homogeneity—equivalent to ID); however, the reproducibility of the latter two was only moderate (DiffEnt) and poor (ID) in our study. In Shafiq-Ul-Hassan et al.’s phantom study [58], 11 texture features panned out as highly stable and defined as percent coefficient of variation (%COV) below 30%. Out of them, the ones directly comparable with the present work are first-order Uniformity (indicated as Energy in [58]); GLCM InvVar and JointAvg; GLRLM GLN and RLN; and NGTDM Coarseness and Strength. Among these only the GLCM ones attained good stability in our experiments, albeit the threshold for ‘goodness’ adopted in [58] (%COV < 30%) was far more generous than can be used here (S≤10%).

One much debated question in radiomics is whether intensity resampling should be absolute or relative [59]. In the first case, the window bounds are determined *a priori* and are invariable across different scans and ROIs, whereas in the second case they are relative to the region of interest. When intensity values represent quantities with the same physical meaning across different scans (such as Hounsfield Units—assuming there are no calibration errors) the use of absolute resampling seems logical [42,60] and this was the decision made here. As detailed in Section 2.2, we determined the window bounds based on the actual distribution of the intensity values in the whole dataset, but other choices such as mediastinal (W:350, L:50) or lung (W:1500, L:−600) window would be reasonable options as well. Notably, our results indicate that changes in intensity quantisation had little consequence on most of the first-order features; whereas the effect on the other classes was generally stronger and with much larger intra-class variability (see Figure 5). This suggests that particular care should be taken in the selection of texture features different than first-order when changes in signal quantisation are involved.

Another methodological point that requires further attention is what figure of merit should be used for assessing feature stability. Althought Intra-class Correlation Coefficient (ICC) is the common practice in the literature [35,37,38,40], we did not think this was the correct choice here. There are two reasons behind this observation. First, ICC assumes a statistical model where the true scores are normally distributed among the study population [61], but of course this is not guaranteed. Second, in a multicentric study much of the inter-subject variance may come from differences in parameters that are hard to control, such as voxel size, slice thickness, tube voltage, etc., all of which may have unknown and unpredictable effects on the estimated ICC. In order to avoid these potential problems we based our evaluation on a direct measurement of intra-rater difference at the nodule level, as for instance used in Varghese et al.’s phantom study [29], and averaged the result over the whole population. The resulting *S* (Equation (1)–(2)) avoids the unpredictable effects consequent to between-nodule differences in the acquisition settings; furthermore, it has a straightforward interpretation (values are bound between 0% and 100%) and does not rely on any assumptions on the distribution of the underlying data.

Focussing on the potential implications of radiomics in clinical decision making, one major problem related to the lack of feature stability is that the results are difficult to reuse across multiple centres. If one centre determines that having a certain feature value above a given threshold is predictive of malignancy in lung nodule screening, a second one can reuse that result only if (a) the features are computed using the same settings or (b) the features are stable enough. Concerning intensity quantisation, of course one sensible approach would be to stick to one value ($N_{g}=256$ is a common choice [16,62,63,64]) in order to have comparable data. However, for some features simple mathematical transformations could be applied to make the features independent of the number of quantisation levels (see for instance Appendix A). In order to avoid or reduce the inter-observer bias related to manual lesion delineation, automated and semi-automated methods offer great promises in terms of speed, accuracy and repeatability [65]. Previous studies have shown that semi-automated segmenters can improve on manual delineation and generate more reproducible radiomics features [37,38].

## 5. Conclusions and Future Work

In recent years, the extraction of quantitative imaging features from lung lesions on CT has attracted increasing research interest as a potential tool to improve diagnosis, risk stratification and the follow-up of lung cancer. Still, the applicability of radiomics across multiple institutions and on large populations of patients depends a great deal on the robustness of the image features to changes in the acquisition settings, preprocessing procedures and lesion delineation methods. In this context the objective of this work was to evaluate the impact of lesion delineation and intensity quantisation on the stability of texture features extracted from suspicious lung nodules on CT scans. Specifically, we assessed the robustness of 88 texture features from six classes: first-order, GLCM, GLDM, GLRLM, GLSZM and NGTDM. For reproducible research purposes, we carried out the experiments on a public dataset of lung nodules (LIDC-IDRI) and employed open-source tools (Python and PyRadiomics) for feature extraction. Implementation settings and code are also available to the public for future comparisons and evaluation.

The results indicate that the impact of changes in lesion delineation and intensity quantisation was important: of the 88 texture features included in the study, only 18 showed good stability (S≤10%) relative to both types of change. These findings suggest caution when it comes to building predictive models involving CT features obtained with different quantisation schemes and/or affected by contour variability. From a clinical standpoint, our results are useful as they identify a set of stable CT texture features that can contribute to the diagnosis of lung cancer. This is very important for the discovery of robust imaging biomarkers that may help characterise lung lesions, particularly, in those cases where the anatomical site or the clinical presentation of the patient rule out other invasive methods (e.g., biopsy).

The present investigation indicates different directions for future research. In confirming previous studies [58], we found that most texture features were sensitive to intensity quantisation of the CT signal. This suggests (a) that the mathematical formulations of these features may need to be revised in order to remove such dependency (as for instance proposed in Appendix A) and/or (b) that the number of quantisation level should be defined/recommended in internationally-accepted guidelines (standardisation). Similarly, the effects of intra-observer and inter-observer variability in lesion delineation could be reduced by recurring to automated and semi-automated segmentation procedures. As pointed out in [35], this is particularly critical in lung cancer where tumour progression is associated with density changes in the core and peri-tumoural region. Hence, the need for radiomics to take into account both areas.

## Figures and Tables

**Table 1:** Complete list (names and abbreviations) of the first-order, GLCM and GLDM features considered in this study. For each class, the features are listed in column-wise alphabetical order.

**First-Order Features**		
Entropy (Entropy)	Inter-quartile range (IQR)	Kurtosis
Mean absolute deviation (MAD)	Maximum (Max)	Mean (Mean)
Median	Minimum (Min)	Range
Robust mean absolute deviation (RMAD)	Standard deviation (Std)	Skewness
Uniformity
**Features from Grey-Level Co-Occurrence Matrix (GLCM)**		
Autocorrelation (Acorr)	Cluster shade (ClShade)	Cluster prominence (ClProm)
Cluster tendency (ClTen)	Contrast (Contr)	Correlation (Corr)
Difference average (DiffAvg)	Difference entropy (DiffEnt)	Difference variance (DiffVar)
Joint average (JointAvg)	Joint energy (JointEnergy)	Joint entropy (JointEntropy)
Informational measure of correlation ‘1’ (IMC1)	Informational measure of correlation ‘2’ (IMC2)	Inverse difference (ID)
Inverse difference moment (IDM)	Inverse difference moment normalised (IDMN)	Inverse difference normalised (IDN)
Inverse variance (InvVar)	Maximal correlation coefficient (MCC)	Maximum probability (MaxProb)
Sum average (SumAvg)	Sum entropy (SumEnt)	Sum of squares (SumSquares)
**Features from Grey-Level Difference Matrix (GLDM)**		
Dependence entropy (DE)	Dependence non-uniformity (DN)	Dependence non-uniformity normalised (DNN)
Dependence variance (DV)	Grey-level non-uniformity (GLN)	Grey-level variance (GLV)
High grey-level emphasis (HGLE)	Large dependence emphasis (LDE)	Large dependence high grey-level emphasis (LDHGLE)
Large dependence low grey-level emphasis (LDLGLE)	Low grey-level emphasis (LGLE)	Small dependence high grey-level emphasis (SDHGLE)
Small dependence low grey-level emphasis (SDLGLE)	Small dependence emphasis (SDE)	

**Table 2:** Complete list (names and abbreviations) of the GLRLM, GLSZM and NGTDM texture features considered in this study. For each class, the features are listed in column-wise alphabetical order.

**Features from Grey-Level Run-Length Matrix (GLRLM)**		
Grey-level non-uniformity normalised (GLNN)	Grey-level non-uniformity (GLN)	Grey-level variance (GLV)
High grey-level run emphasis (HGLRE)	Long-run emphasis (LRE)	Long-run high grey-level emphasis (LRHGLE)
Long-run low grey-level emphasis (LRLGLE)	Low grey-level run emphasis (LGLRE)	Run entropy (RE)
Run-length non-uniformity normalised (RLNN)	Run-length non-uniformity (RLN)	Run percentage (RP)
Run variance (RV)	Short-run emphasis (SRE)	Short-run high grey-level emphasis (SRHGLE)
Short-run low grey-level emphasis (SRLGLE)
**Features from Grey-Level Size-Zone Matrix (GLSZM)**		
Grey-level non-uniformity (GLN)	Grey-level non-uniformity normalised (GLNN)	Grey-level variance (GLV)
High grey-level zone emphasis (HGLZE)	Large area emphasis (LAE)	Large area high grey-level emphasis (LAHGLE)
Large area low grey-level emphasis (LALGLE)	Low grey-level zone emphasis (LGLZE)	Size-zone non-uniformity (SZN)
Size-zone non-uniformity normalised (SZNN)	Small area emphasis (SAE)	Small area high grey-level emphasis (SAHGLE)
Small area low grey-level emphasis (SALGLE)	Zone entropy (ZE)	Zone percentage (ZP)
Zone variance (ZV)
**Features from Neighbouring Grey-Tone Difference Matrix (NGTDM)**		
Busyness	Coarseness	Complexity
Contrast	Strength	

**Table 3:** Assessment of the stability of texture features against lesion delineation (A) and intensity resampling (B): experimental settings.

Scenario	Quantisation Levels ($N_{g}$)	Lesion Delineation
A	256	Four diff. observers
B	{32,64,128,256}	50% consensus

**Table 4:** Qualitative grading of feature stability and related colourmap based average on sMAPE.

Range	Qualitative Label
0%≤S≤5%	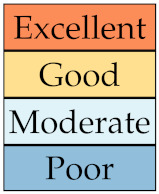
5%<S≤10%	
10%<S≤20%	
20%<S≤100%	

**Table 5:** Stability of the first-order features against lesion delineation and intensity resampling. *S* indicates average sMAPE (Equation (2)).

Class	Feature Name/Abbreviation	*S*	
		Delineation	Resampling
First-order	Max	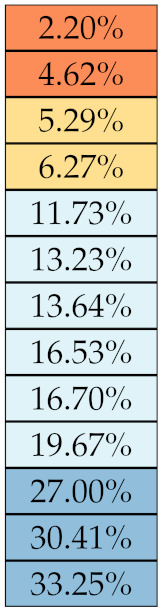	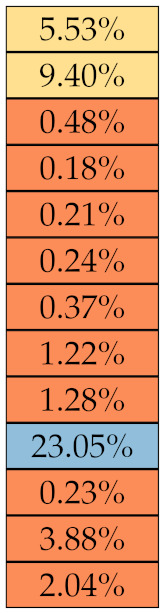
	Entropy		
	Range		
	Min		
	Std		
	MAD		
	Kurtosis		
	IQR		
	RMAD		
	Uniformity		
	Mean		
	Median		
	Skewness		

**Table 6:** Stability of the GLCM features against lesion delineation and intensity resampling. *S* indicates average sMAPE (Equation (2)).

Class	Feature Name/Abbreviation	*S*	
		Delineation	Resampling
GLCM	IDMN	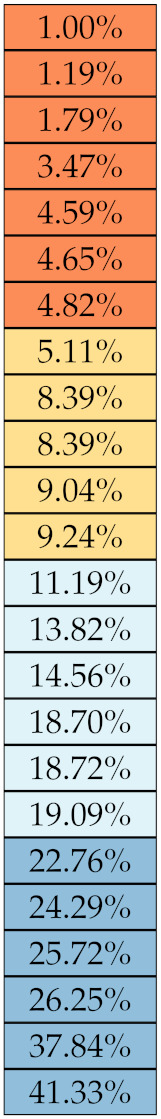	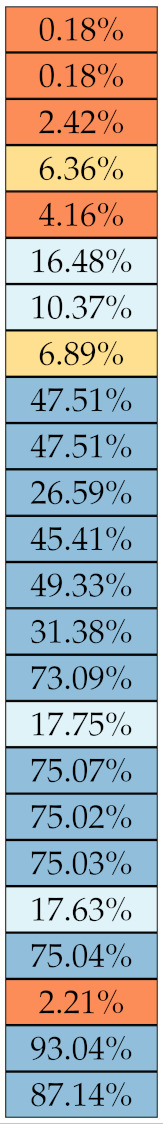
	IDN		
	IMC2		
	MCC		
	JointEntropy		
	IMC1		
	DiffEnt		
	SumEnt		
	SumAvg		
	JointAvg		
	ID		
	InvVar		
	DiffAvg		
	IDM		
	Acorr		
	JointEnergy		
	DiffVar		
	Contrast		
	SumSquares		
	MaxProb		
	ClTen		
	Correlation		
	ClProm		
	ClShade		

**Table 7:** Stability of the GLDM features against lesion delineation and intensity resampling. *S* indicates average sMAPE (Equation (2)).

Class	Feature Name/Abbreviation	*S*	
		Delineation	Resampling
GLDM	DE	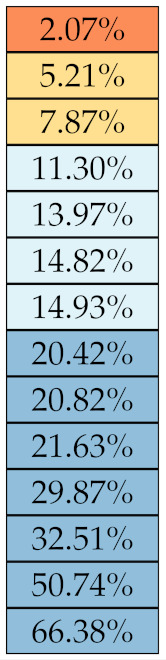	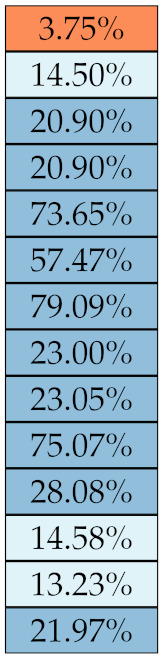
	SDE		
	DNN		
	DN		
	HGLE		
	LDHGLE		
	SDHGLE		
	LDE		
	GLN		
	GLV		
	DV		
	SDLGLE		
	LGLE		
	LDLGLE		

**Table 8:** Stability of the GLRLM features against lesion delineation and intensity resampling. *S* indicates average sMAPE (Equation (2)).

Class	Feature Name/Abbreviation	*S*	
		Delineation	Resampling
GLRLM	SRE	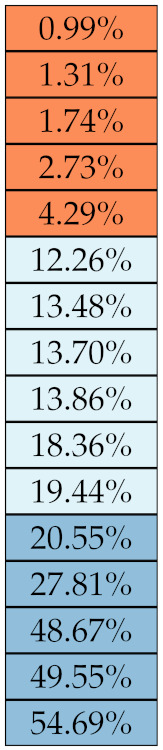	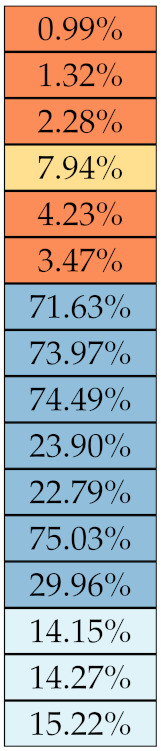
	RP		
	RLNN		
	RE		
	LRE		
	RLN		
	LRHGLE		
	HGLRE		
	SRHGLE		
	GLNN		
	GLN		
	GLV		
	RV		
	SRLGLE		
	LGLRE		
	LRLGLE		

**Table 9:** Stability of the GLSZM features against lesion delineation and intensity resampling. *S* indicates average sMAPE (Equation (2)).

Class	Feature Name/Abbreviation	*S*	
		Delineation	Resampling
GLSZM	SAE	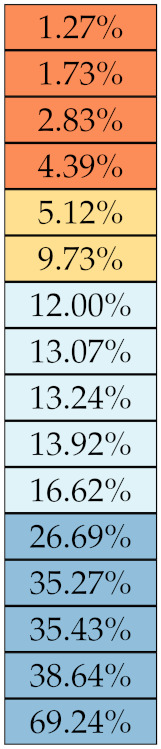	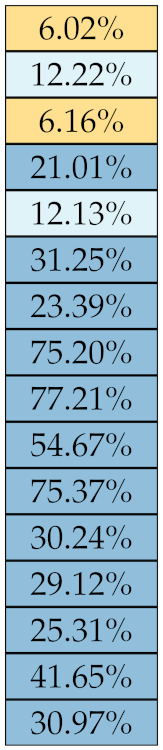
	SZNN		
	ZE		
	GLN		
	ZP		
	GLNN		
	SZN		
	HGLZE		
	SAHGLE		
	LAHGLE		
	GLV		
	LAE		
	SALGLE		
	LGLZE		
	ZV		
	LALGLE		

**Table 10:** Stability of the NGTDM features against lesion delineation and intensity resampling. *S* indicates average sMAPE (Equation (2)).

Class	Feature Name/Abbreviation	*S*	
		Delineation	Resampling
NGTDM	Strength	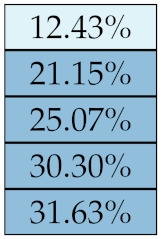	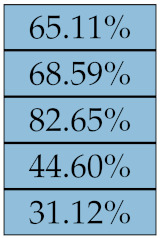
	Contrast		
	Complexity		
	Busyness		
	Coarseness		

## Data Availability

The data presented in this study are openly available in the Lung Image Database Consortium image collection (LIDC-IDRI) at http://doi.org/10.7937/K9/TCIA.2015.LO9QL9SX, accessed on 29 May 2021.

## Abbreviations

The following abbreviations are used in this manuscript (For texture features abbreviations please refer to Table 1 and Table 2).

ACS	American Cancer Society
CCC	Concordance Correlation Coefficient
COV	Coefficient of Variation
CT	Computed Tomography
CXRs	Chest radiographies
GLCM	Grey-level Co-occurrence Matrix
GLDM	Grey-level Difference Matrix
GLRLM	Grey-level Run Length Matrix
GLSZM	Grey-level Size-zone Matrix
ICC	Intra-class Correlation Coefficient
HU	Hounsfield Units
MRI	Magnetic Resonance Imaging
NGTDM	Neighbouring Grey-tone Difference Matrix
ROI	Region of Interest
sMAPE	Symmetric Mean Absolute Percentage Error
PET	Positron Emission Tomography

## Appendix A. Proposal for Normalised Formulations of First-Order Entropy and Uniformity

The formulations of Entropy and Uniformity currently adopted in the literature [42,55] are by definition dependent on the number of intensity levels $N_{g}$. We have the following:

(A1) $$\mathrm{Entropy}=-\sum_{i=1}^{N_{g}}\log_{2}\left[p\left(i\right)\right]$$

(A2) $$\mathrm{Uniformity}=\sum_{i=1}^{N_{g}}{\left[p\left(i\right)\right]}^{2}$$

where $p\left(i\right)$ represents the probability of occurrence of the *i*-th intensity level. The following are normalised formulations with values in [0,1].

(A3) $$\mathrm{Normalised}\;\mathrm{Entropy}=-\frac{1}{\log_{2}\left(N_{g}\right)}\sum_{i=1}^{N_{g}}\log_{2}\left[p\left(i\right)\right]$$

(A4) $$\mathrm{Normalised}\;\mathrm{Uniformity}=\frac{N_{g}}{1-N_{g}}\left\{\sum_{i=1}^{N_{g}}{\left[p\left(i\right)\right]}^{2}-\frac{1}{N_{g}}\right\}$$
